# Strong Anharmonicity at the Origin of Anomalous Thermal Conductivity in Double Perovskite Cs_2_NaYbCl_6_

**DOI:** 10.1002/advs.202305861

**Published:** 2023-12-18

**Authors:** Antonio Cappai, Claudio Melis, Daniela Marongiu, Francesco Quochi, Michele Saba, Francesco Congiu, Yihui He, Tyler J. Slade, Mercouri G. Kanatzidis, Luciano Colombo

**Affiliations:** ^1^ Department of Physics University of Cagliari Cittadella Universitaria Monserrato (CA) 09042 Italy; ^2^ Department of Chemistry Northwestern University 2145 North Sheridan Road Evanston IL 60208 USA

**Keywords:** anharmonicity, anomalous thermal transport, double halide perovskite

## Abstract

Anomalous thermal transport of Cs_2_NaYbCl_6_ double‐halide perovskite above room temperature is reported and rationalized. Calculations of phonon dispersion relations and scattering rates up to the fourth order in lattice anharmonicity have been conducted to determine their effective dependence on temperature. These findings show that specific phonon group velocities and lifetimes increase if the temperature is raised above 500 K. This, in combination with anharmonicity, provides the microscopic mechanism responsible for the increase in lattice thermal conductivity at high temperatures, contrary to the predictions of phonon transport theories based on solely cubic anharmonicity. The model accurately and quantitatively reproduces the experimental thermal conductivity data as a function of temperature.

## Graphical abstract

An anomalous non‐monotonic thermal conductivity trend in Cs_2_NaYbCl_6_ perovskite above 500 K has been measured and rationalized via first‐principles phonon calculations. Besides the occurrence of significative coherent thermal transport effects, fourth‐order anharmonicity has been identified as the crucial mechanism responsible for hardening of acoustic phonons with temperature, ultimately leading to a corresponding thermal conductivity increase.

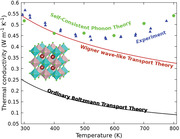

## 1 Introduction

Thermal conductivity is a crucial property of materials, especially for thermoelectric generation. Converting temperature differences in electrical power achieves maximum conversion efficiency when employing materials with large electrical conductivity to flow electricity yet low thermal conductivity to maintain large temperature differences between the two poles of the device. An important research focus to advance thermoelectric generators (TEGs) is therefore to uncover low‐thermal‐conductivity materials that are earth abundant, benign, and easy to fabricate.

Double halide perovskites have emerged as promising materials^[^ ^1^ ^]^ thanks to their recently observed ultralow thermal conductivity.^[^ ^2^ ^]^ The unit cell of typical double perovskite materials is realized by two metal cations, with valences of respectively +1 (e.g., Na or Ag) and +3 (e.g., In, Bi, or a rare earth element), two monovalent Cs cations (or an organic molecule, like methylammonium CH_3_NH_3_) and six halides (typically Cl or Br). The double halide framework supports, therefore a large variety of possible combinations, with room for optimization of material properties.

However, thermal conductivity is a function of temperature, κ(*T*), and thus varies according to the temperature interval TEGs operate in. The understanding of heat conduction based on harmonic lattice vibrations, also known as phonon transport theory, forecasts that κ(*T*)∝*T* ^−α^ (with α ⩾ 1), due to phonon scattering increasing with temperature, resulting in reduced thermal conductivity. Nevertheless, significant variations from such a dependence have been recently observed in double perovskites and attributed either to lattice anharmonicity or to disorder and lattice defects, or even to grain boundaries and glassy behavior.^[^ ^3^, ^4^, ^5^, ^6^ ^]^ Identifying the most promising materials and optimizing their properties requires understanding the true origin of such anomalous thermal conductivity.

In this work, we selected double perovskite Cs_2_NaYbCl_6_ and synthesized it both as powder and in the form of a single crystal to focus on the intrinsic anharmonicity effects on thermal conductivity and rule out defects and grain boundaries. We measure a sharp violation of the *T* ^−α^ law, with κ(*T*) *increasing* in the temperature range 500~K ⩽ *T* ⩽ 800~K. In order to rationalize the observed anomaly in thermal conductivity, we combine state‐of‐the art electronic and crystal structure calculations with the most advanced computational methods to retrieve thermal conductivity. A fully ab initio investigation has been carried out on the structural and lattice dynamical properties of Cs_2_NaYbCl_6_, and its high‐temperature thermal transport properties have been determined. The effective relationship between temperature, phonon dispersion relations, and scattering rates has been determined by calculating them up to the fourth order in lattice anharmonicity using the improved self consistent phonon theory as implemented by Tadano et al.^[^ ^7^ ^]^ This analysis revealed that specific phonon group velocities and lifetimes increased with increasing temperature above 500 K. Calculations demonstrated that the observed anomalous thermal conductivity is a result of the amplified phonon contributions combined with lattice anharmonicity, contradicting the expectations of standard phonon transport theory. Moreover, the quantitative calculations aligned completely with the experimental data.

## 2 Methods

### 2.1 Theory

A fully atomistic picture, relying on a microscopic determination of the thermal conductivity, must be developed to provide a rationale for the experimentally observed trends. More specifically, in order to take into account the observed (see below) deviations from the *T* ^−α^ law, an extension of the paradigmatic approach based on the solution of linearized Boltzmann transport equation (LBTE), is needed. At the state of the art, several different theoretical approaches have been developed and applied to this aim, at different levels of sophistication depending on the theoretical strategy adopted. These methods span from the generalization of constitutive transport equations in order to take into account possible phonon–phonon interference phenomena^[^ ^8^, ^9^ ^]^ to the direct introduction of temperature‐related effects by renormalization of phonon frequencies.^[^ ^7^, ^10^ ^]^

In the first case, a Wigner transport equation^[^ ^9^ ^]^ is worked out, able to describe within a unique theoretical framework either the phonon‐like thermal transport (typical of weakly anharmonic crystals where heat carriers propagate and scatter as particles) and the wave‐like one (typical of disordered media, like glasses). Interestingly enough, the Wigner formulation is as well able to cope with an intermediate situation where particle‐like and wave‐like phenomena occur simultaneously.

The fundamental result emerging from this approach is the modification of the standard expression used for the evaluation of thermal conductivity. In the standard approach, where phonons are assumed to scatter as corpuscular entities, the Peierls–Boltzmann (PB) expression for the six independent components of the lattice thermal conductivity tensor ${\overset{↔}{\kappa }}^{\mathrm{PB}}$ is

(1) $$\kappa_{\alpha \beta }^{\mathrm{PB}}=\frac{1}{N_{q}V}\sum_{qj}c_{qj}v_{qj,\alpha }v_{qj,\beta }\tau_{qj}$$

where the summation is over all phonon branches *j* and q points in the first Brillouin zone (in total as many as *N*_*q*_). Here, $c_{qj}=\hbar \omega_{qj}\partial n_{qj}/\partial T$ is the mode heat capacity, where $n_{qj}$ is the population of the (q,j) phonon mode. In Equation (1), the $\omega_{q,j}$ eigenfrequencies and the α, β components of the group velocity $v_{qj,\alpha }$ are obtained in a pure harmonic approximation, while the $\tau_{qj}$ lifetimes are calculated by using perturbation theory. Only three‐phonon processes are usually taken into account for the calculation of $\tau_{qj}$, since it is expected that they play the major role in thermal transport phenomena.^[^ ^11^ ^]^

By contrast, Wigner transport equation dictates a new expression for thermal conductivity, hereafter referred to as ${\overset{↔}{\kappa }}^{W}$. If only third order phonon scattering processes are taken into account and energy‐renormalization effects are explicitly neglected, Wigner thermal conductivity is expressed as

(2) $$\begin{array}{c} \kappa_{\alpha \beta }^{W}=\kappa_{\alpha \beta }^{\mathrm{PB}}+\frac{1}{N_{q}V}\sum_{q,j\neq j^{\prime }}\frac{c_{qj}\omega_{qj^{\prime }}+c_{qj^{\prime }}\omega_{qj}}{\omega_{qj}+\omega_{qj^{\prime }}}v_{qjj^{\prime }}^{\alpha }v_{qj^{\prime }j}^{\beta }\frac{\Gamma_{qj}+\Gamma_{qj^{\prime }}}{{\left(\omega_{qj}-\omega_{qj^{\prime }}\right)}^{2}+{\left(\Gamma_{qj}+\Gamma_{qj^{\prime }}\right)}^{2}} \end{array}$$

where $\Gamma_{qj}={\left(2\tau_{q,j}\right)}^{-1}$ is the total phonon linewidth of phonon mode qj. The $v_{qjj^{\prime }}$ term represents^[^ ^9^, ^12^ ^]^ the generalization of group velocity taking into account the coupling between phonon branches.

As pointed out elsewhere,^[^ ^9^ ^]^ thermal conductivity derived from the Wigner transport equation differs from the usual PB expression for the presence of the additional term named *coherent thermal conductivity*. However, in both PB and Wigner formulations, the temperature dependence of κ_αβ_ is only exploited through the $c_{qj}$ terms, while phonon frequencies, velocities, and lifetimes are rigorously calculated at zero temperature (harmonic approximation). Renormalization approaches, instead, are built on different foundations.^[^ ^10^ ^]^ In this case, a pure particle‐like description of phonons is adopted: accordingly, Equation (1) must be used to stick to this assumption. The role of anharmonicity, however, is here treated with a rather different approach. In the original PB formulation, anharmonicity only affects lifetimes: this holds, however, only provided that anharmonicity is sufficiently small to be treated as a perturbation to the pure harmonic description of the system. In other words, anharmonicity (usually truncated at third order) is only responsible for introducing a finite lifetime of the pure harmonic energy levels, which, in turn, are considered to be unaffected. This approximation is too restrictive when anharmonicity is very strong, as it will be shown to be in the present case, since the shift of energy levels induced by high order anharmonicity cannot longer be disregarded. As a matter of fact, the possibility to explore regions of the potential energy surface (PES) where high‐order anharmonicity terms are no longer negligible determines a more significant role of temperature in determining the phonon spectrum. More specifically, modifications of phonon frequencies and lifetimes result from temperature‐induced changes in interatomic force constants (IFCs): To this aim, even if Equation (1) is still applied, the temperature dependence is naturally cast in the form

(3) $$\kappa_{\alpha \beta }^{\mathrm{SCPH}}=\frac{\hbar }{N_{q}V}\sum_{qj}\omega_{qj}\left(T\right)v_{qj,\alpha }\left(T\right)v_{qj,\beta }\left(T\right)\tau_{qj}\left(T\right)\frac{\partial n(q,j)}{\partial T}$$

where we explicitly used a notation stressing the fact that temperature dependence is now directly hard‐coded in phonon eigenfrequencies, velocities, and lifetimes.

In order to rigorously treat the frequency shifts and lifetime modifications induced by temperature, a class of theories, grouped collectively under the name of *self‐consistent phonon* (SCP) theories, has been developed so far. In this paper, we will focus on the formulation implemented by Tadano et al.^[^ ^13^ ^]^ in the Alamode code,^[^ ^7^ ^]^ naturally stemming from the first modelizations of anharmonic lattice dynamics using statistical Green's function methodologies.^[^ ^14^, ^15^, ^16^, ^17^, ^18^, ^19^, ^20^, ^21^ ^]^ Referring to ref. [10] for details, it is here sufficient to highlight the fact that by following the SCPH approach the role of anharmonicity is treated at two levels: i) introduction of a temperature‐dependent phonon lifetime and ii) shift of the phonon energy compared to the case of a non‐interacting phonon. In other terms, to take into account all the modifications that a non‐interacting (bare) phonon undergoes by interacting (via anharmonicity) with all the remaining ones, the concept of propagator $G_{qjj^{\prime }}\left(\omega \right)$ is introduced as a descriptor of the causal correlation between the creation and destruction of a phonon with q momentum and *j*, *j*′ starting and final polarization, respectively. It can be proved^[^ ^20^ ^]^ that the interacting (dressed) phonon propagator $G_{qjj^{\prime }}\left(\omega \right)$ can be expressed in terms of the bare phonon propagator $G_{qjj^{\prime }}^{0}\left(\omega \right)$ and an additional term, called phonon self‐energy $\Sigma_{qjj^{\prime }}\left(\omega \right)$, via the Dyson equation

(4) $$G_{qjj^{\prime }}{\left(\omega \right)}^{-1}=G_{qjj^{\prime }}^{0}{\left(\omega \right)}^{-1}+\Sigma_{qjj^{\prime }}\left(\omega \right)$$

where the *G* ^0^(ω) is the Green function describing a non‐interacting phonon

(5) $$G^{0}\left(\omega \right)=-\frac{2\omega_{qj}}{\omega^{2}-\omega_{qj}^{2}}\delta_{jj^{\prime }}$$

while the self‐energy $\Sigma_{qjj^{\prime }}\left(\omega \right)$ can be in principle calculated to include anharmonic effects at any order. The self‐energy $\Sigma_{qjj^{\prime }}$ contains all the information about the role of anharmonicity, since its imaginary part is directly related to the phonon lifetime, while its real part accounts for the energy shifts induced by phonon–phonon interactions.^[^ ^19^ ^]^

As reported in ref. [13], the presence of these energy shifts can be alternatively treated in terms of renormalized phonon frequencies, critically depending on the mean square displacements via fourth‐order interatomic force constants. Interestingly enough, this introduces the temperature‐dependence in both phonon frequencies and group velocities, as reported in Equation (3).

### 2.2 Computational Protocol

Density functional theory (DFT) calculations were performed in periodic boundary conditions as implemented in the Quantum Espresso suite.^[^ ^22^ ^]^ The generalized gradient approximation of Perdwe, Burke, and Ernzerhof (PBE)^[^ ^23^ ^]^ was used in all calculations. Ultrasoft pseudopotential (USPP)^[^ ^24^ ^]^ was used in combination with a plane‐wave basis set to model the electronic structure. A plane‐wave and charge density cutoff of 66 and 323 Ry, respectively, were adopted in all calculations, in combination with energy and force convergence criteria of 10^−10^~Ry and 10^−9^~Ry~Å ^−1^, respectively. For the sole purpose of speeding up self‐consistent field (SCF) convergence, a Gaussian 10^−4^~Ry smearing was adopted.

Structural relaxation of the primitive unit cell (ten atoms) of Cs_2_NaYbCl_6_ was performed using a Γ centered 8 × 8 × 8~*k*‐point mesh in order to validate the cubic lattice geometry and estimate the lattice parameter.

In order to calculate the thermal conductivity according to Equations (1)–(3), all the phonon‐related quantities are evaluated starting from the interatomic force constants (IFCs). In particular, harmonic IFCs are needed in all cases to evaluate the phonon frequencies and velocities, while cubic IFCs are used to evaluate the lifetimes. Only in the case of the SCPH method, fourth order constants are also necessary in order to properly renormalize the phonon frequencies as outlined above.

We adopted the finite displacement method, as implemented in the Alamode suite^[^ ^7^ ^]^ to calculate the IFCs. To this aim, a conventional cubic cell containing 40 atoms was adopted: A cutoff of 5.29 and 4.23 Å was imposed to generate the displacement patterns for the evaluation of the third and fourth order anharmonic IFCs, while all possible interactions within the 40 atoms cell were taken into account in evaluating the harmonic IFCs. The generation of displacement patterns was treated systematically for the evaluation of harmonic and cubic IFCs, while for fourth order IFCs the compressive sensing technique^[^ ^25^, ^26^ ^]^ was adopted in combination with the elastic net fitting algorithm,^[^ ^27^ ^]^ an approach allowing to reduce the otherwise overwhelming computational cost required for a systematic treatment of such high‐order anharmonicity.

Finite size artifacts were excluded by evaluating harmonic and cubic IFCs in a supercell obtained as 2 × 2 × 2 replica of the primitive cell, with an increased cutoff for cubic IFCs of 87.31 Å: we thus adopted the conventional cubic cell for all following analysis.

Force calculations on each displaced atomic configuration were performed by adopting the lattice parameter resulting from full structural relaxation without altering the plane‐wave and charge density cutoffs and with a sampling density in *k*‐space of 0.15 Å^−1^.

By a fitting procedure of the force–displacement data sets the harmonic, cubic, and quartic IFCs were obtained and used to calculate the phonon dispersion curves, lifetimes, and corrections to phonon dispersion curves, respectively. At this stage, the thermal conductivity was calculated accordingly to the expressions in Equations (1)–(3) using the PB, Wigner, and SCPH approaches, respectively, since the Alamode code offers all three possibilities. A 5 × 5 × 5~*q*‐point mesh was found to be sufficiently dense to assure the convergence of all the six κ_αβ_ values. The bulk thermal conductivity κ_bulk_ was finally estimated all the cases as the trace of the thermal conductivity tensor as

(6) $$\kappa {\left(T\right)}_{\mathrm{bulk}}=\frac{1}{3}\left[\kappa {\left(T\right)}_{xx}+\kappa {\left(T\right)}_{yy}+\kappa {\left(T\right)}_{zz}\right]$$

this procedure was repeated for all three possible calculations according to the PB, Wigner, and SCPH approaches. The mode Grüneisen parameter $\gamma_{q,j}$ was finally calculated as

(7) $$\gamma_{q,j}=-\frac{V}{\omega_{q,j}}\frac{d\omega_{q,j}}{dV}$$

and next used to investigate the mode‐specific anharmonicity features.

In order to further verify the validity of the computational protocol here adopted, the bulk modulus *B*_0_ was estimated by fitting the SCF energy *E*(*a*) as a function of the lattice parameter *a* using the Birch–Murnaghan equation of state (EOS). In the case of a cubic lattice with equilibrium lattice parameter *a*_0_, the EOS can be cast in the form

(8) $$E\left(a\right)-E_{0}=\frac{9}{16}\xi^{3}a^{3}B_{0}\left[{\left(\xi^{2}-1\right)}^{3}B_{0}^{\prime }+2{\left(\xi^{2}-1\right)}^{2}\left(3-2\xi^{2}\right)\right]$$

where *E*_0_ is the SCF evaluated at equilibrium lattice constant, $B_{0}^{\prime }={\left(\partial B/\partial p\right)}_{p=0}$, and ξ = *a*_0_/*a*. Finally, in order to test the accuracy of calculated IFCs, specific heat capacity $C_{v}\left(T\right)$ was calculated accordingly to the microscopic formulation

(9) $$C_{v}\left(T\right)=\frac{k_{B}}{N_{q}}\sum_{q,j}{\left(\frac{\hbar \omega_{qj}}{2k_{B}T}\right)}^{2}{\mathrm{cosech}}^{2}\left(\frac{\hbar \omega_{qj}}{k_{B}T}\right)$$

where ω_*qj*_ are properly corrected to include finite temperature effects via SCPH approach.

## 3 Results and Discussions

### 3.1 Crystal Structure and Stability

**Figure 1:**
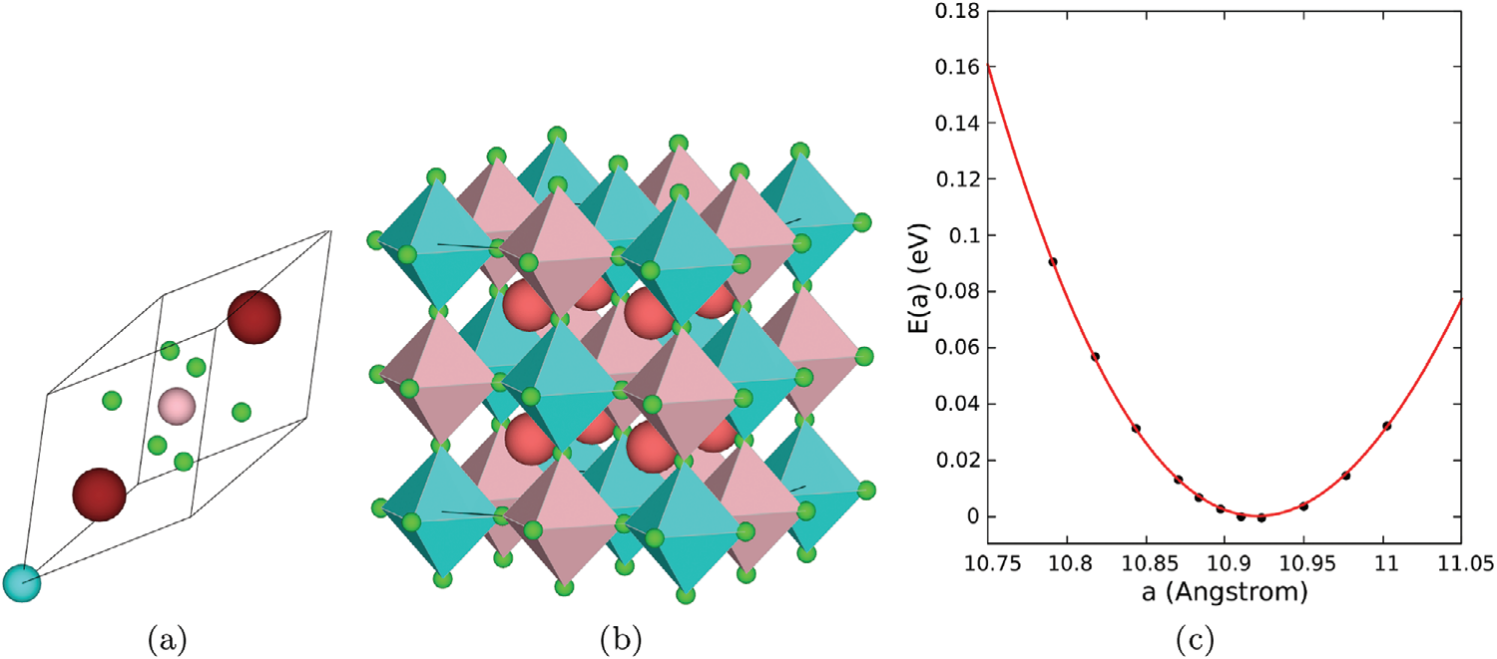
a) Primitive unit cell for Cs_2_NaYbCl_6_ obtained from full structural optimization. The equilibrium lattice parameters are *a* = *b* = *c* = 7.709~Å and α = β = γ = π/3. b) 40 atoms conventional cubic cell obtained by remapping the ten atoms primitive cell. The conventional cubic cell was used for harmonic and anharmonic vibrational‐related properties. The bulk modulus *B*_0_ is obtained by fitting the DFT energies obtained at different lattice parameters using the Birch–Murnaghan equation of state, resulting in *B*_0_ = 16.28~GPa.

The results of full structural relaxation of Cs_2_NaYbCl_6_ primitive unit cell (**Figure** **1**a) are reported in **Table** **1**. Starting from a trigonal structure as an initial guess, the lattice parameters *a* and α were found to converge at 7.709~Å and 60°, respectively.

**Table 1:** Comparison of lattice parameters and angles for Cs_2_NaYbCl_6_ obtained from DFT calculations and Birch–Murnaghan estimate resulting from fit of total DFT energy as a function of lattice parameter. Experiment value at 300 K is reported in the last column.

	DFT	Birch–Murnaghan	Experiment
*a* = *b* = *c*	7.709 Å	7.717 Å	7.547 Å
α = β = γ	60°	60°	60°

In order to check the presence of a possible tilting of the octahedral constitutive units, commonly found in halide and double halide perovskites,^[^ ^3^, ^5^, ^28^ ^]^ the trigonal unit cell was used to generate a conventional cubic cell containing 40 atoms (see Figure 1b), where a total of eight octahedral units were present. A full relaxation of such a large cell proved the stability of the cubic phase, with an equilibrium lattice constant of *a*_0_ = 10.92~Å, and the absence of any tilting. This result is in line with the calculation of the Goldschmidt tolerance parameter *t* ^[^ ^29^, ^30^ ^]^

(10) $$t=\frac{r_{A}+r_{X}}{\sqrt{2}\left(r_{B}+r_{X}\right)}$$

where *r*_A_, *r*_B_, and*r*_X_ are the ionic radii of Cs, Yb, and Cl, respectively. The *t* value is 0.966, within the interval 0.9 ⩽ *t* ⩽ 1.0, for which the perovskite can exist in a stable cubic phase. Following the recent suggestion of Bartel et al.,^[^ ^31^ ^]^ the *t* parameter was also recalculated accordingly to the new definition proposed, re‐assuring the stability of cubic phase. Experimental data in the temperature range 300~K ⩽ *T* ⩽ 800~K confirm our predictions, with a lattice constant at 300K equal to $a_{0}^{\exp}=10.671\;Å$.

**Figure 2:**
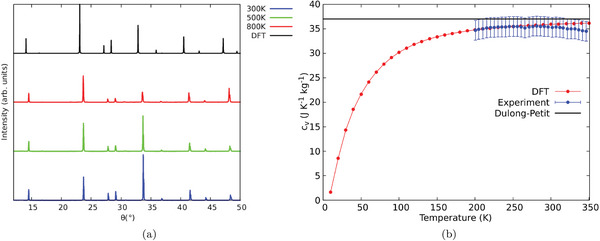
a) DFT calculated (black line) and experimental X‐ray diffraction pattern of Cs_2_NaYbCl_6_ at 300 K (blue line), 500 K (green line), and 800 K (red line). b) Calculated (red) versus measured (blue) specific heat capacity: the black line represents the Dulong–Petit limit.

To assess the validity of the computational protocol adopted, the X‐ray diffractogram calculated on the cubic conventional cell using the VESTA^[^ ^32^ ^]^ routine was compared with the corresponding XRD experimental data obtained with copper K_α_ line (λ = 1.54059~Å). As shown in **Figure** **2**a, the simulated DFT X‐ray diffractogram was found to be in agreement with the experiment in the whole temperature range investigated. Moreover, the analysis of the diffractogram allows us to rule out the occurrence of defects and glassy structure. Experimental XRD at 300, 500, and 800 K also allow to dismiss the possibility of a structural phase transition, since the overall diffractogram structure is only marginally affected by the temperature variation. Starting from the fully relaxed conventional cubic cell, the bulk modulus was obtained by fitting the dependence *E*(*a*) (see Figure 1c) of the SCF energy on the lattice parameter *a*. To this aim the Birch–Murnaghan equation of state^[^ ^33^, ^34^ ^]^ was used, and a value of 16.28~GPa was obtained, in line with the typical values of this class of perovskites.^[^ ^35^, ^36^ ^]^

In the framework of quasi‐harmonic approximation,^[^ ^37^ ^]^ we also calculated the thermal expansion coefficient by performing 16 phonon dispersions calculations, each one with a different volume in the range 1225–1420 Å ^3^. As reported in **Table** **2**, we found a fair agreement with the experimental data here taken from XRD measurements. The values results of DFT calculations and the corresponding measured values are in line with the typical ones observed for this class of materials and sufficiently small to justify the use of a fixed cell in the calculation of *T*‐dependent phonon properties.

**Table 2:** Comparison of thermal expansion coefficient α as resulting from measurements of XRD data (α_exp._) and from DFT calculations (α_DFT_) in the framework of quasi‐harmonic approximation.

*T*	300 K	400 K	500 K	650 K	800 K
α_exp._ (· 10^−5^K^−1^)	1.8 ± 0.1	1.65 ± 0.09	1.49 ± 0.05	1.25 ± 0.06	1.02 ± 0.13
α_DFT_ (· 10^−5^K^−1^)	1.8 ± 0.2	1.9 ± 0.1	1.88 ± 0.17	1.54 ± 0.17	1.6 ± 0.2

As a final test for the accuracy of calculated IFCs, the results for heat capacity calculation $C_{v}\left(T\right)$ as a function of temperature *T* provided the data shown in Figure 2b. A good agreement with experimental data is found, giving another sound assessment for the IFCs computational setup.

### 3.2 Harmonic and Anharmonic Vibrational Properties

**Figure 3:**
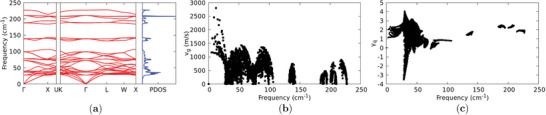
a) Calculated phonon dispersion curves and phonon density of states (PDOS) for Cs_2_NaYbCl_6_. Finite displacements to extract interatomic force constants were performed on 80 atoms supercell obtained as 2 × 2 × 2 of the primitive cell, with the equilibrium lattice constant (*a*_0_ = 10.896~Å). b) Phonon group velocity spectrum. c) Gruneisen parameter γ_*q*_ as a function of the phonon mode frequency.

**Figure 4:**
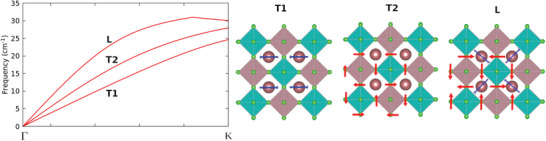
The three acoustic phonon modes along the Γ → K direction: blue (red) arrows indicate the corresponding displacements of Cs (Cl) atoms.

The calculated phonon dispersion curves are shown in **Figure** **3**a, with the corresponding phonon group velocities represented in Figure 3b: In both figures, the *q*‐point path was chosen along the high symmetry directions of the first Brillouin zone. From direct inspection of the eigenvectors of the dynamical matrix, it was found that the lowest vibrational modes are associated with Cl and Cs vibrations, as shown in **Figure** **4** where, for sake of clarity, just the acoustic branches are plotted. Here the longitudinal acoustic (L) and the two transverse acoustic (T1 and T2) branches are represented along the Γ − K direction, where T1 and T2 are not degenerate. In Section 3.3, we will provide evidence that such Cs‐ and Cl‐related acoustic motions as well as Cl‐related optical modes are directly related with the anomalous thermal conductivity increase for *T* > 500~K.

The analysis of the phonon density of states (PDOS) and the phonon group velocities revealed a close similarity with the corresponding values found by Pandey et al.^[^ ^38^ ^]^ in their work on ultralow thermal conductivity in lead‐free perovskites. It can be noted that in both cases the PDOS are characterized by a relatively broad distribution, with a strong peak at around ≈45~cm^−1^. In Cs_2_NaYbCl_6_, however, sharper peaks appear at the higher frequencies associated with optical modes, mainly related to oscillation of Cl atoms.

Phonon group velocities frequency spectrum displays, as well, significant deviations from the above‐mentioned paradigmatic cases. High phonon group velocities can be in fact observed in an almost uniform distribution in the 0~cm^−1^ ⩽ ν ⩽ 100~cm^−1^ frequency range, with a mean value of the order of ≈1800~m~s^−1^. This datum is in agreement with the ultralow thermal transport evidence in lead‐free perovskites.^[^ ^38^ ^]^

Finally, the inspection of the Gruneisen parameter as a function of the phonon frequency (Figure 3c) reveals the signature of a clear and strong anomaly in the frequency region 25~cm^−1^ ⩽ ν ⩽ 40~cm^−1^, where it reaches negative values as large as −3.5. The occurrence of negative values of Gruneisen parameter is well documented in literature, for silicon^[^ ^39^ ^]^ as well as both inorganic and organic systems^[^ ^40^, ^41^, ^42^ ^]^ and usually associated to uncommon phenomena as negative expansion coefficients (NEC). In our case, even if no evidence of NEC was found, we attribute the anomalous trend of γ_*q*_ to the strong role of anharmonicity, which we included as a correction to the harmonic phonon dispersion curves by using SCPH.

### 3.3 Finite‐Temperature Effects on Thermal Transport

**Figure 5:**
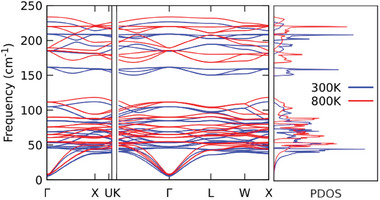
Calculated temperature‐dependent phonon dispersion curves and phonon density of states (PDOS) at 300 K (blue) and 800 K (red) for Cs_2_NaYbCl_6_.

**Figure 6:**
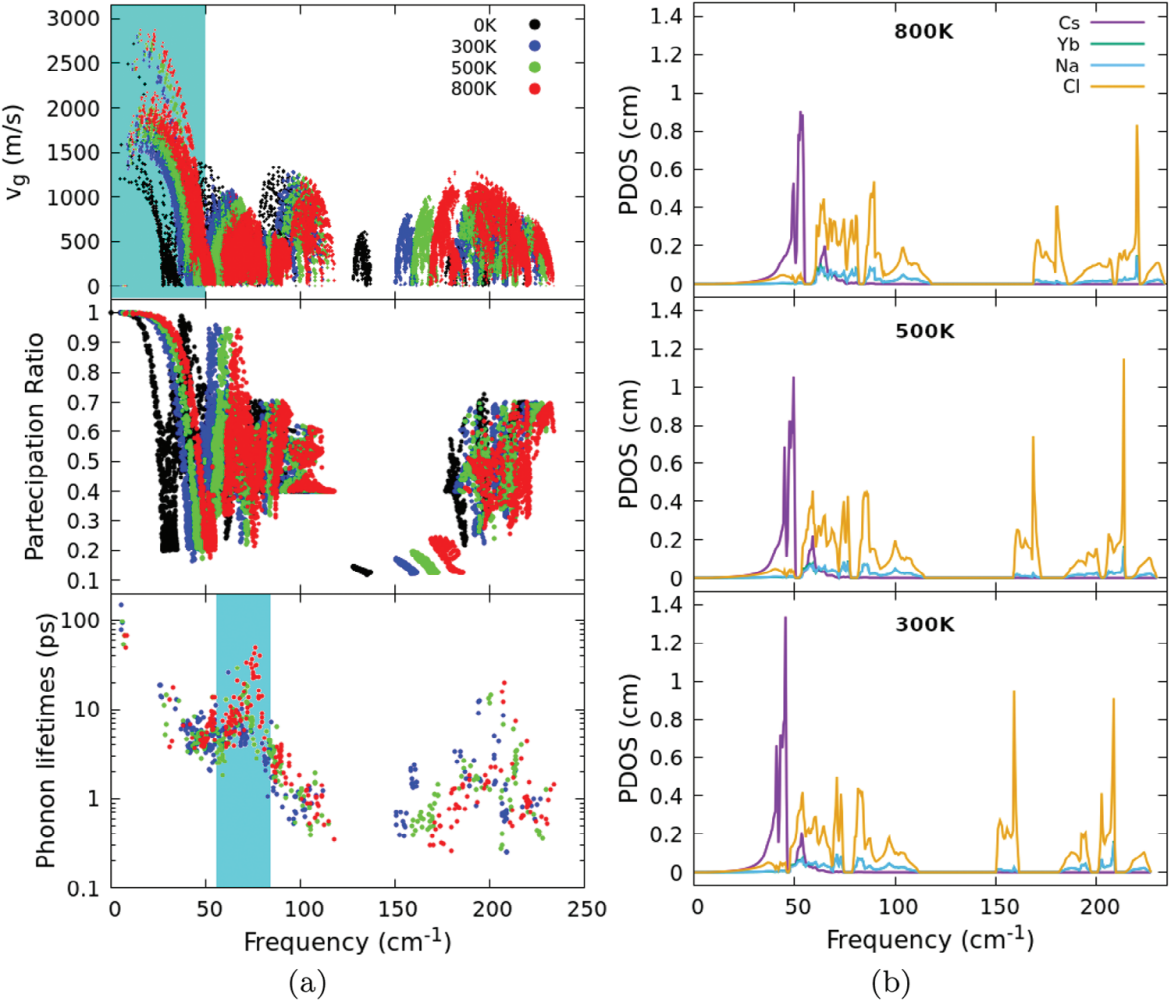
a) Phonon group velocity spectrum (top panel), participation ratio (middle panel), and phonon lifetimes (bottom panel) of Cs_2_NaYbCl_6_ at 0 K (black) and for the three finite temperatures here investigated, namely 300 K (blue), 500 K (green), and 800 K (red). Cyan regions evidence the frequency ranges where anomalous increases of phonon group velocities and lifetimes are more evident. b) Projected atomic‐phonon density of states (PDOS) at 300, 500, and 800~K. The dominant role of Cs‐ and Cl‐atoms in determining vibrational properties is evidenced as well as the dominant role of Cs‐atoms in low frequency region.

In **Figure** **5** we compare the renormalized phonon dispersion curves and phonon density of states (PDOS) at the lowest and highest finite temperatures investigated, namely 300 and 800 K, while the corresponding phonon group velocities, participation ratio and phonon lifetimes are shown at zero and three finite temperatures in **Figure** **6**a (top panel), 6a (middle panel), and 6a (bottom panel), respectively. Dispersion curves in Figure 5 exhibit a sizeable frequency shift by increasing temperature. In particular, two remarkable effects are observed: i) a large shift of optical modes with ν ⩾ 120~cm^−1^ toward higher frequencies, as shown in the PDOS representation in Figure 5 and ii) an increase in the steepness of the acoustic modes. Phonon group velocities at the three target temperatures here investigated are presented in Figure 6a (top panel), where an increase of up to ≈50% can be observed for acoustic modes when temperature is raised from 0 to 300 K. In the frequency range 50~cm^−1^ ⩽ ν ⩽ 100~cm^−1^, the dominant feature is the shift in frequency, with velocity values remaining stable around ≈1200~m~s^−1^. Conversely, in the frequency range where the anomalous increase of phonon group velocities occurs, mainly involving Cl and Cs vibrational modes. The dominant role of these two atomic species in determining the thermal transport mechanism is evidenced by the atom‐projected phonon density of states (PDOS) at 300, 500, and 800~K, represented in Figure 6b: two particular features are evident, namely i) the presence of a large peak at low frequency related to the Cs atomic motions and ii) the broad extension in frequencies of the Cl‐related signal. As the temperature is increased, anharmonicity‐induced frequency shift is observed, as well as the increase in extension of Cl contributions, which also appear more noisy at 800~K.

**Figure 7:**
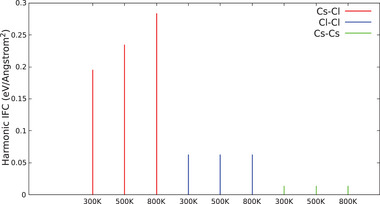
Averaged interatomic force constants (IFCs) for Cs–Cl, Cl–Cl, and Cs–Cs bonds at 300, 500, and 800 K. Values are in line with reported values in literature.^[^ ^43^ ^]^

We speculate that this anomaly is a result of a significant modification of their chemical bonding with the surrounding atoms. We argue that such bonds undergo stiffening when temperature is increased, an effect that we attribute to the specificity of the atomic orbitals involved as well as to the peculiar crystalline environment in which Cs and Cl are embedded. Such a peculiar bond hardening with temperature is clearly observed by analyzing the magnitude of the harmonic interatomic force constants (IFCs) at 300, 500, and 800 K (see **Figure** **7**). Such a non‐standard behavior is strictly related to the anomalies observed in phonon lifetime, resulting in a global non‐monotonic dependence of thermal conductivity with temperature.

The participation ratio shown in Figure 6 (middle panel) reveals the effect of temperature on promoting the delocalization of low‐frequency phonons. The rise in temperature appears to largely impact mainly the phonon modes with frequencies below 50~cm^−1^, while higher frequency modes are less affected. A similar trend is observed in the evolution of phonon lifetimes shown in Figure 6 (bottom panel), where an intriguing counter‐intuitive behavior appears for phonons with frequencies 50~cm^−1^ ⩽ ν ⩽ 100~cm^−1^: As a matter of fact their lifetimes increase with temperature, reaching values similar to those ones of low‐lying acoustic modes at *T* = 800~K. This anomaly is, however, limited to this frequency range, as the expected decrease with temperature of the lifetime is observed for acoustic modes, with a ≈20% reduction observed when *T* is increased from 300 to 800 K. A similar trend is found in the higher frequency domain, where a tenfold decline is evident but is superimposed with the phonon energy renormalization frequency shift.

By carefully analyzing the displacement patterns for phonons in this very limited frequency range, we realize that they are largely related to Cl atom motions (which, remarkably, do not exhibit any anomaly in phonon velocity behavior as *T* increases). Since, as it can be easily seen in Equation (3), the dependency of κ(*T*) on phonon velocities is quadratic and linear in lifetime, we thus expect that the anomaly due to Cs and Cl atom motions (affecting phonon velocities) is the most dominant feature underlying the experimentally observed anomaly in κ(*T*). Ultimately, we identify the root for this anomaly in the strong anharmonicity of Cs and Cl bonds in the double halide perovskite here examined.

**Figure 8:**
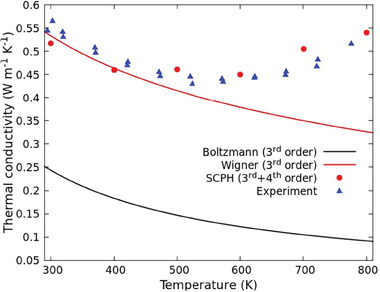
Cs_2_NaYbCl_6_ thermal conductivity κ(*T*) as a function of the temperature. The black and red lines depict the theoretical prediction of κ(*T*) calculated with the Peierls–Boltzmann (cubic anharmonicity, Equation (1)) and Wigner approach (cubic anharmonicity, Equation (2)). The anomalous increase in the high temperature regime, as experimentally observed (blue triangles), is eventually reproduced by inclusion of fourth‐order anharmonicity using SCPH approach (Equation (3), red circles).

The impact of such anharmonicity effects is crucial in determining the lattice thermal conductivity, as shown in **Figure** **8**, where κ(*T*) is calculated according to the Peierls–Boltzmann formulation (black line), the Wigner formulation including the coherent thermal conductivity component (red line), and SCPH approach (red circles). Experimental data collected as explained in Section 2.1 are as well shown by blue triangles.

A first interesting result is represented by the severe underestimate of κ(*T*), even at room temperature, when the PB approach is used, while it is found that both the Wigner and SCPH approaches successfully recover the experimental datum. This observation confirms the need to go beyond the standard PB approach. In that case the large underestimate of κ(*T*) can be attributed to the large role played by anharmonicity in this material, which cannot be treated as only a perturbative effect at the third order. The physical interpretations of this phenomenon, however, depend on the actual superior approach adopted. According to the SCPH theory, where a clean particle‐like picture is adopted, the agreement with the experimental data is provided by the joint effect of i) global phonon frequency shift, and ii) selected modification of phonon group velocities and lifetimes (see discussion above). Eventually, the overall effect is the increase in calculated κ(*T*) with respect to the pure harmonic case (as shown in Figure 8). In particular, we argue that the inclusion of fourth‐order anharmonicity in the description of the potential energy is crucial, being the principal cause for frequencies shifts.

Similarly, the Wigner approach, treating the anharmonicity as the source of tunnelling of phonons belonging to different branches, is able to solve the large underestimate obtained when the PB approach is used. This is a clear indication that the coherent component of thermal conductivity in Cs_2_NaYbCl_6_ accounts for a fraction as large as 54% of the overall thermal conductivity observed at *T* = 300~K. Nevertheless, above *T* = 500~K the Wigner approach predicts that κ(*T*) monotonically decreases as κ(*T*) ≈ *T* ^−0.55^, contrary to the experimental observations.

In capturing this subtle effect, the role of renormalization of phonon eigenfrequencies, induced by fourth‐order anharmonicity, appears to be essential. In conclusion, the SCPH approach successfully reproduces this feature by highlighting the anomalous increase of phonon velocities in a specific frequency range.

**Figure 9:**
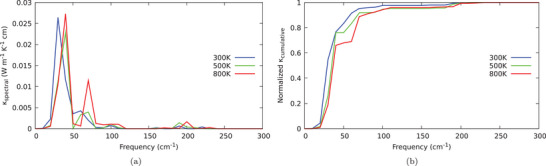
a) Spectral thermal conductivity; b) normalized thermal conductivity calculated according to SCPH theory at three different temperatures.

To conclude our analysis of the impact of anahrmonicity on thermal conductivity, the spectral and normalized cumulative thermal conductivity are represented in **Figure** **9**a,b, respectively: over the full temperature range, we found that the major contributions to κ are basically related to Cl‐ and Cs‐atoms, consistent with the analysis of atom‐projected PDOS. In particular, the contribution of Cs‐atoms appears to account for ≈50% of the overall thermal conductivity, quickly reached for frequencies below ≈30~cm^−1^, while a non‐negligible fraction of the remaining thermal conductivity contribution is basically due to Cl‐atomic motions, which are in turn the most affected by frequency shift due to anharmonicity

## 4 Conclusions

We have conducted an experimental synthesis and characterization of Cs_2_NaYbCl_6_ double perovskite, observing an anomalous thermal conductivity as a function of temperature. Our DFT investigation focused on the experimentally observed violation of the κ(*T*) ≈ *T* ^−|α|^ law, since a clear increase is observed in the temperature range 500–800 K. After validating the computational protocol adopted and reproducing the observed structural properties of the double perovskite, we calculated phonon group velocities and lifetimes, including their temperature dependence due to interatomic potential anharmonicity. Our findings revealed an anomalous increase with temperature of group velocities and lifetimes for phonon with 50 ⩽ ν ⩽ 100~cm^−1^. The observed phonon hardening represents a noticeable variation from the commonly observed softening, as expected for the majority of crystals. This phenomenon has been recently shown in other perovskite materials, such as BaZrO_3_ (as reported by Zheng et al.^[^ ^44^ ^]^), and lead‐free halide perovskites (by Pandey et al.^[^ ^38^ ^]^). These two studies clearly observed a non‐negligible phonon hardening trend with increasing temperature, pointing out that this effect, while atypical, is not uncommon within perovskite systems. The essential role of renormalizing phonon eigenfrequencies, caused by fourth‐order anharmonicity, which is currently absent in the existing theoretical framework of the Wigner approach, becomes apparent in detecting this subtle yet noticeable anomaly. The SCPH approach effectively demonstrates this characteristic by highlighting the abnormal rise in phonon velocities within a particular frequency range. By solving the Boltzmann transport equation in the relaxation time approximation, we found that this unexpected trend is at the origin of the κ(*T*) anomalous behavior, which we interpreted as the consequence of the interplay between lattice dynamics and thermal transport in perovskites due to the presence of fourth‐order anharmonicity.

Moreover, the Wigner approach proved to be correct in recovering the observed value at 300 K but not in describing the anomaly above 500 K, which, in our understanding, is strictly related to the alteration of phonon speeds and frequencies.

Overall, we attribute the observed anomalous thermal conductivity increase for *T* ⩾ 500~K to the stiffening of acoustic branches, due fundamentally to Cs and Cl atomic motions. The stiffening of the chemical bonds involved, in our perspective, is somewhat very unusual to be observed and can only be explained in terms of a strong anaharmonic character of the interaction, arising from an interplay between the specificity of the atomic orbitals involved as well as the peculiar and complex crystalline structure where electrons are constrained.

## 5 Experimental Section

Powder of Cs_2_NaYbCl_6_ was synthesized by dissolving 10 mmol of Yb_2_O_3_ in 100 mL of hot hydrochloric acid (37%) under vigorous stirring, then 20 mmol of NaCl, 40 mmol of CsCl, and 50 mL of H_2_O were added and left to react for 10 min. The powder was collected after solvent evaporation under low pressure. A cylindrical single crystal of Cs_2_NaYbCl_6_ was grown in a vertical Bridgmann furnace by melting the as‐synthesized Cs_2_NaYbCl_6_ powder at 880 °C in a carbonized quartz tube and then slowly cooling down. The crystallization process was obtained with the quartz tube descending at a rate of 1mm h^−1^ until 700 °C followed by a slow temperature decrease until room temperature. A 5.91 mm large and 1.43 mm thick polished disk was obtained from the as‐grown crystal with a crystal cutting saw and used for thermal diffusivity measurements with the Laser Flash Analysis (LFA‐457, Netzsch) thermal diffusivity measurement system. The temperature ranged from room temperature to 500 °C under a nitrogen atmosphere.

Powder X‐ray diffraction measurements were performed on finely ground crystals with a Bruker D8Advance diffractometer equipped with Cu‐*k*α radiation and a PSD detector. Variable temperature X‐ray diffraction patterns were also recorded between room temperature and 530 °C. Measurements were recorded with a 0.025° step size over the 2θ angle range of 12°–60°. The heat capacity at constant pressure was measured using the heat capacity option of a Quantum Design physical properties measurement system (PPMS DynaCool) in the temperature range 200–350 K. The sample, a 17.4 mg fragment, was mounted onto the sample platform using a thin layer of Apiezon H grease to optimize thermal coupling between the sample and calorimeter stage. The heat capacity option of the PPMS used a thermal relaxation technique. Each measurement cycle at a certain temperature consisted of a heating period and a cooling period, followed by a fitting of the entire temperature response of the sample platform to a model that accounted for both the thermal relaxation of the sample platform to the bath temperature and the relaxation between the sample platform and the sample itself.^[^ ^45^ ^]^ The measurement was performed on warming with 5 K temperature intervals, and three data points were taken at each temperature and averaged to give more accurate results. The contribution to the heat capacity from the grease was subtracted from the total measured heat capacity to yield the heat capacity of the sample.

## Conflict of Interest

The authors declare no conflict of interest.

## Data Availability

The data that support the findings of this study are available from the corresponding author upon reasonable request.
